# The erectile and ejaculatory implications of the surgical management of rectal cancer

**DOI:** 10.1111/iju.15235

**Published:** 2023-06-26

**Authors:** Armin Ghomeshi, John Zizzo, Raghuram Reddy, Joshua White, Aden Swayze, Sanjaya Swain, Ranjith Ramasamy

**Affiliations:** ^1^ Herbert Wertheim College of Medicine Florida International University Miami Florida USA; ^2^ University of Miami Miller School of Medicine Miami Florida USA; ^3^ Desai Sethi Urology Institute, University of Miami Miller School of Medicine Miami Florida USA

**Keywords:** abdominoperineal resection, colorectal cancer, ejaculatory dysfunction, erectile dysfunction, low anterior resection, sexual dysfunction

## Abstract

Colorectal cancer is a significant cause of cancer‐related deaths worldwide. Although advances in surgical technology and technique have decreased mortality rates, surviving patients often experience sexual dysfunction as a common complication. The development of the lower anterior resection has greatly decreased the use of the radical abdominoperineal resection surgery, but even the less radical surgery can result in sexual dysfunction, including erectile and ejaculatory dysfunction. Improving the knowledge of the underlying causes of sexual dysfunction in this context and developing effective strategies for preventing and treating these adverse effects are essential to improving the quality of life for postoperative rectal cancer patients. This article aims to provide a comprehensive evaluation of erectile and ejaculatory dysfunction in postoperative rectal cancer patients, including their pathophysiology and time course and strategies for prevention and treatment.

**Abbreviations & Acronyms**
APR: abdominoperineal resection
cAMP: cyclic adenosine monophosphate
cGMP: cyclic guanosine monophosphate
CRC: colorectal cancer
EMG: electromyography
ICIs: intracavernosal injections
IPP: inflatable penile prosthesis
LAR: lower anterior resection
NCCN: National Comprehensive Cancer Network
PANP: Pelvic autonomic nerve preservation
PGE1: prostaglandin E1
PVS: penile‐vibro stimulation
SoR: space of Retzius

## INTRODUCTION

Colorectal cancer is the second‐highest cause of cancer‐related mortality in the United States, with over 147 000 new cases and 53 000 deaths reported in 2020 alone.^1^ Advancements in surgical technology and technique have significantly improved outcomes for colorectal cancer (CRC) patients over the years.^2^ However, improved survival has made quality of life (QoL) and functional outcomes much more relevant. For male CRC survivors, erectile function and ejaculation remain a frequent source of concern.^2^ Sexual dysfunction has long been recognized as an operative complication. Radical abdominoperineal resection (APR) was a revolutionary surgical approach, with curative potential.^2^ The development of the lower anterior resection (LAR) has markedly decreased the popularity of APR in recent years, with studies reporting an APR to LAR ratio between 1:3 and 1:4.^3^ Although LAR data suggest a better prognosis and lower recurrence than APR, sexual function is still affected, and studies have shown that 64% of patients experience a significantly decreased quality of sexual life.^3^, ^4^

The continued persistence of postoperative sexual dysfunction is a matter of concern, given the profound impact that sexual dysfunction can have on one's quality of life. A more thorough understanding of the underlying characteristics of these complications may provide insight into effective strategies for preventing and treating these adverse effects and thus improving the QoL for postoperative CRC patients. This article aims to provide a comprehensive evaluation of erectile and ejaculatory dysfunction in postoperative CRC patients. We will discuss the pathophysiology and strategies for preventing and treating these adverse effects.

## PHYSIOLOGY OF ERECTION

An erection of the penis occurs when both the penile arteries dilate and the erectile tissue (corpus cavernosum and corpus spongiosum) relax.^5^ The dilation of the penile arteries results in increased blood flow to the penis raising both the penile volume and intrapenile pressure, with the latter being an accurate measurement of penile erection. Chemical messengers including nitric oxide carried facilitate the synthesis, and prevent degradation, of two intracellular second messengers, cyclic nucleotides guanosine monophosphate (cGMP) and adenosine monophosphate (cAMP).^6^, ^7^ Unlike other visceral tissues, such as the gut and uterus, the penis does not have an intrinsic autonomic innervation or spontaneous muscle contractions. Instead, the penis is supplied with nerve fibers both autonomically, including sympathetic and parasympathetic fibers, and somatically, including sensory and motor fibers.^5^ The sympathetic and parasympathetic nerves originate from neurons in the spinal cord and peripheral ganglia, and come together to form the cavernous nerves.^7^

The primary driver of penile tumescence is parasympathetic stimulation although a reduction in sympathetic nervous system activity also contributes. The parasympathetic supply to the penis originates from the S2–S4 sacral segments.^8^ However, patients with sacral spinal cord injury individuals can still achieve erections through psychological stimulation, although they may not be as rigid. Erections resulting from psychological stimulation are not possible in patients with injuries above T9, indicating that the main cause is the central suppression of sympathetic stimulation.^9^, ^10^ Individuals with injuries above T9 may still experience reflexogenic erections, demonstrating that the preservation of the sacral reflex arc, which is responsible for penile stimulation‐mediated erections is the primary mechanism behind reflexogenic tumescence.^11^, ^12^

## PHYSIOLOGY OF EJACULATION

The organs involved in ejaculation have a rich supply of autonomic nerves, both sympathetic and parasympathetic, from the pelvic plexus. This plexus is retroperitoneal to the rectum, and posterolateral to seminal vesicle.^13^ It receives signals from the hypogastric and pelvic nerves, as well as the caudal paravertebral sympathetic chain.^14^ The sympathetic nerves release neurotransmitters such as norepinephrine and acetylcholine.^15^ The integration of signals from genital stimulation occurs at the sacral spinal level to produce emission of ejaculate. The preganglionic sympathetic nerve cells responsible for ejaculation are located in the intermediolateral cell column and the central autonomic region of the thoracolumbar segments (T12‐L1).^16^ The sympathetic fibers connect to the pelvic plexus via hypogastric nerve.^17^ The preganglionic parasympathetic nerve cells are located in the sacral parasympathetic nucleus and connect to the pelvic plexus via the pelvic nerve.^18^ Onuf's nucleus, located in the sacral spinal cord, projects fibers through the motor component of the pudendal nerve, thus leading to contraction of the pelvic muscles and ejaculation.^19^

## SURGERIES FOR RECTAL CANCER AND IMPACTS ON SEXUAL FUNCTION

The goal of CRC surgery is to remove the tumor with a minimum margin of 2 cm for a low rectal tumor that involves preserving the sphincter or 5 cm for a rectosigmoid upper rectal tumor.^19^ LAR involves restoring the continuity of the intestine through an anastomosis (Table 1). A thorough lymph node dissection, including evaluation of the mesorectum, is performed through harvesting the sigmoid mesentery and mesorectum.^19^ TME is necessary for tumors in the middle and lower rectum to reduce the risk of local recurrence.^20^ The goal is to remove the rectal tumor and pararectal lymph nodes while preserving structures outside the rectal fasciation, such as nerve fibers that innervate the bladder and prostate.^21^

**TABLE 1:** Comparison of abdominoperineal resection (APR) and low anterior resection (LAR) for rectal cancer management.

	APR	LAR
Features	Involves anterior abdominal and perineal incisions Removal of the distal colon, rectum, anal sphincter complex, and anus with the creation of a permanent colostomy Warranted if adequate negative distal margin is not feasible with a sphincter‐sparing procedure Less commonly performed	Involves removal of the rectum through abdominal incisions with coloanal anastomosis Preserves the anal sphincter More commonly performed Preferred if a negative distal margin is feasible
Indications	Tumor location: distal (lower) 1/3 of the rectum	Tumor location: proximal (upper) 2/3 of the rectum
Surgical outcomes	Higher circumferential margin involvement rates Higher local recurrence rates^a^ Poorer prognosis	Lower circumferential margin involvement rates Lower local recurrence rates^a^ Better prognosis
Advantages	Suitable for patients with poor preoperative anorectal function	Less invasive Preserved bowel‐anus continuity Shorter hospital lengths of stay Lower OR time
Disadvantages	Permanent colostomy More invasive Longer hospital lengths of stay Longer OR time	Age and anorectal function barriers Anastomotic leak risk
Quality of Life (incl. sexual dysfunction)	Generally worse^b^	Generally better^b^

a While multiple studies on patients after APR for rectal cancer have shown higher local recurrence rates than after LAR,^4^, ^26^, ^27^ other studies have found no difference.^28^, ^29^

b Quality of life metrics in patients after undergoing LAR for rectal cancer have largely been shown to be superior to those noted in patients after undergoing APR.^30^ However, differing results have been reported.^28^, ^31^, ^32^

**FIGURE 1:**
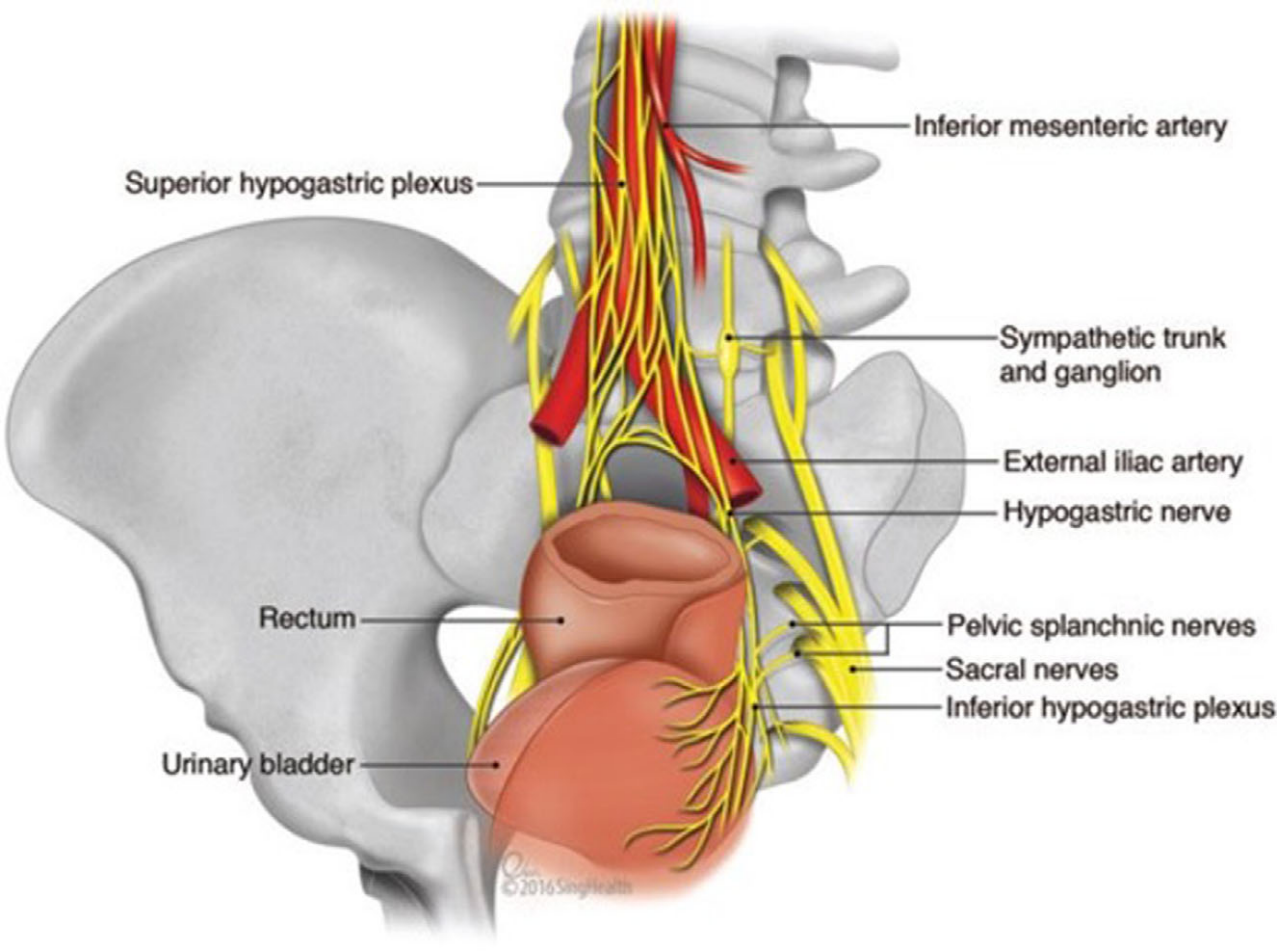
Anatomy of the pelvic autonomic nerves with relation to rectum.^22^ Source: Chew et al., 2016, figure 1. Reproduced with permission of Oxford University Press.

**FIGURE 2:**
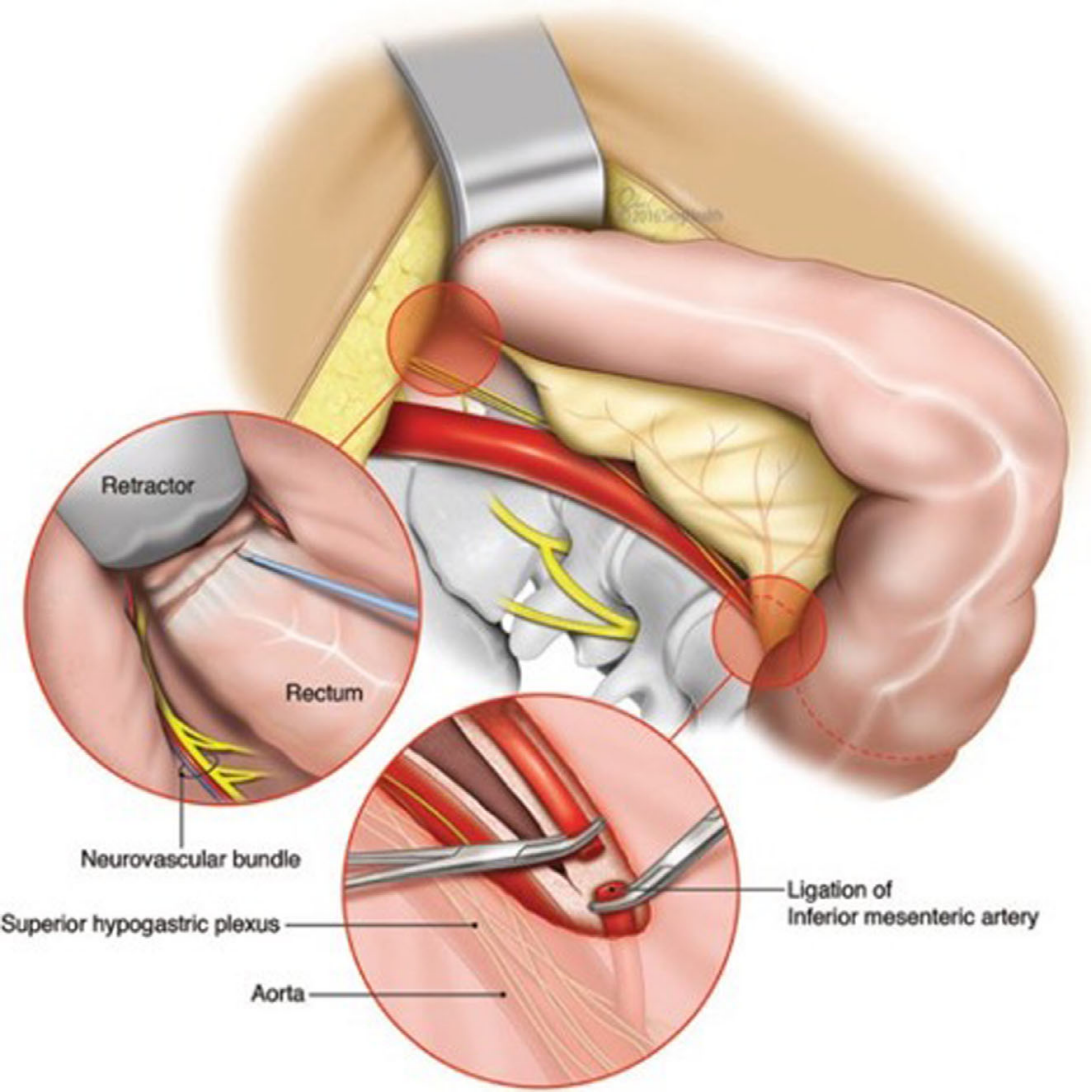
The relationship of the rectum and pelvic autonomic nerves during open surgery.^22^ Source: Chew et al., 2016, figure 3. Reproduced with permission of Oxford University Press.

Injury to the autonomic nerves may occur during high ligation of the inferior mesenteric artery (Figure 1).^22^ The superior hypogastric nerves, located near the sacral promontory and presacral region, can also be affected (Figure 2).^22^ Injuries to the sympathetic nerves can cause retrograde ejaculation. The pelvic splanchnic nerves are situated near the lateral stalks and middle hemorrhoidal artery in the posterolateral area of the pelvis, and these can be damaged during rectal dissection or by excessive pulling.^23^ If the sympathetic nerves are damaged in this area, it may impact the patient's ability to ejaculate, while injury to the parasympathetic nerves may cause erectile dysfunction. There is also a risk of damage near the seminal vesicles and prostate, which contain mixed parasympathetic and sympathetic nerves, which could lead to erectile impotence.

## SEXUAL DYSFUNCTION FOLLOWING SURGERY

Multiple studies demonstrate the detrimental impacts of the surgical treatment of colorectal cancer. The incidence of sexual dysfunction post‐LAR can vary from 32% to 69%.^24^, ^25^, ^26^ Men may be able to regain sexual function over time, as the incidence of sexual dysfunction decreased from 75% (3 months postoperatively) to 55% (9 months postoperatively).^27^ Similar data were seen in a prospective study comparing erectile and ejaculatory function 3 months versus 1 year following surgery.^28^ More data are needed to elucidate the mechanistic causes of the improvements in sexual parameters over time. Additional studies can illuminate possible approaches to accelerate the recovery of erectile and ejaculatory function.

It is important to examine the prevalence of sexual function between various surgical modalities in order to determine the optimal treatment protocol for colorectal patients and their sexual desires. A retrospective study found 32% of patients who underwent APR reported sexual dysfunction, with 18% reporting complete impotence.^29^ On the other hand, complete impotence was seen in any patients who received a LAR.^29^ The study found no correlation between postoperative sexual dysfunction and age, tumor classification, size, location, or perineal wound infection. APR is a more invasive procedure that removes the anal sphincter, indicated for patients with advanced tumors, and thus may explain the increased sexual dysfunction seen with this procedure. However, sexual dysfunction is still seen in LAR, and should not be ignored.

Out of the 12 patients who underwent LAR surgery, 4 reported impotence and 3 reported no ejaculation.^29^ The study found that a higher percentage of sexual dysfunction occurred after abdominoperineal resection than anterior resection (66% vs. 50%). However, the rate was similar for low and very low anterior resection (58% and 66%, respectively).^29^ Older age and very low resection were identified as the two key factors impacting sexual dysfunction after major rectal surgery. Careful operating techniques with skilled surgeons may reduce sexual function‐related complications. Overall, other patient parameters such as BMI, tumor stage, adjuvant chemotherapy, and body image can have profound impacts on sexual function.^29^

Determining when and to what extent sexual dysfunction caused by rectal cancer surgery can resolve or improve is of utmost importance in properly counseling patients on the risks and benefits of various approaches. It is important to note that postoperative sexual dysfunction may be influenced by surgical approach as well as non‐surgical factors, including the use of radiotherapy, aging, comorbidities, and psychological components.^30^, ^31^ Several prospective studies have examined the QoL in patients before and after surgery for rectal cancer to evaluate whether sexual dysfunction and other complications are temporary or permanent. Specifically, studies have shown worsened sexual function (e.g., International Index of Erectile Function [IIEF] scores, ejaculatory dysfunction, libido, dyspareunia, sexual satisfaction, etc.) after rectal cancer resection at 3 months,^28^, ^32^, ^33^, ^34^ 6 months,^32^, ^33^, ^35^, ^36^ and 1 year^28^, ^37^, ^38^, ^39^, ^40^, ^41^ compared to baseline measurements. Potency was found to be regained at 2 years in one study^29^ although others found no improvement at 3 years^42^ or 8 years post‐op.^43^ Thus, long‐term QoL follow‐up studies are mixed and lack consistency. This overlies the need for an individualized discussion with patients surrounding the reality that sexual function may decline, may recover after an extended period, or may never improve following rectal cancer surgery.^31^

*Note: The studies mentioned above recorded QoL in patients with no interventions (e.g., sildenafil, penile rehabilitation, etc.) applied. See “Treatment” section for further information on treatment approaches and the resulting effect on postoperative sexual function.

## PREVENTION STRATEGIES AND SURGICAL CONSIDERATIONS

### Pelvic autonomic nerve preservation

As mentioned previously, rectal surgery‐related sexual dysfunction is suspected to be correlated with the extent of pelvic sympathetic and parasympathetic nerve damage; it is seen in both men and women.^44^ Pelvic autonomic nerve preservation (PANP) was first proposed in the 1980s–1990s due to the high rates of sexual dysfunction seen in conventional techniques. PANP involves utilizing a comprehensive technique to preserve targeted nerve structures and an intact mesorectal fascia, as described by Kim et al.^45^ PANP can be performed in both laparoscopic and robotic‐assisted resection approaches. Studies comparing TME alone versus TME with PANP have shown significant preservation of sexual function with comparable local recurrence and survival rates.^36^, ^39^, ^46^ However, studies have shown higher rates of sexual dysfunction when pelvic lymph node dissection is performed, despite the use of PANP.^47^, ^48^ Thus, PANP remains an integral technique in preserving sexual function without affecting surgical outcomes.

### Intraoperative nerve monitoring

Intraoperative nerve monitoring (IONM) is used in many surgical fields to avoid procedure‐related nerve damage. This typically involves the use of electrodes applied to specific areas, allowing for the measurement and creation of electrical impulses. The impulses provide immediate feedback to the surgeon, allowing insight into neurological changes taking place and for action to be taken to avoid or minimize operative injury. In 2013, a case–control study by Kneist et al. found that open TME with IONM and simultaneous bladder and internal anal sphincter electromyography (EMG) was safe and associated with lower rates of sexual dysfunction.^49^ Zhou et al. demonstrated similar results in patients with low rectal cancer who underwent laparoscopic surgery and IONM.^50^ Further, Schiemer et al. reported the in situ use of fully robot‐guided neuromapping in three patients with low rectal cancer who underwent TME.^51^ The NEUROS trial showed improved functional outcomes in 189 rectal cancer patients undergoing TME with pelvic IONM compared to TME alone, with similar postoperative mortality, operating times, and intraoperative complication rates.^52^ However, barriers to the widespread use of IONM include a lack of well‐defined and proven methodologies and the need for comprehensive equipment and surgical application training.^53^ Nonetheless, pelvic IONM represents a promising development in the quest to reduce sexual dysfunction and improve QoL in patients after rectal cancer surgery.

### Surgical techniques

Over the past several decades, a significant shift toward minimally invasive surgical techniques has ensued for rectal cancer resection. This paradigm shift has succeeded in improving surgical outcomes; however, the question of QoL measures and sexual function remains.^22^ Several studies have shown mixed sexual functioning results in laparoscopic versus open techniques, with some reporting equivalent sexual function rates,^54^ some suggesting quicker potency recovery after laparoscopic resection,^55^, ^56^ and others showing worsened sexual function with laparoscopic technique.^57^, ^58^ The COLOR II randomized trial showed no differences in sexual dysfunction between the laparoscopic and open groups.^59^ Regarding conventional versus robotic‐assisted laparoscopic technique, several studies have shown earlier restoration of erectile function in robotic‐assisted groups compared to laparoscopic.^60^, ^61^, ^62^ However, the ROLARR trial involving 471 patients found no significant difference in sexual dysfunction rates between groups.^63^ Thus, there appears to be no clear‐cut answer regarding which surgical technique best preserves sexual function to date, given the mixed results. As such, selecting a patient's surgical approach should be performed in the context of their tumor location/characteristics, anatomy, and the surgeon's experience. Regardless of technique, caution should be taken to preserve autonomic nerve function whenever possible.

### Radiotherapy

Radiotherapy for rectal cancer has been shown to be independently associated with sexual dysfunction over the past few decades. Such complications are thought to be dose‐dependent and increase with time.^40^, ^64^, ^65^, ^66^ Current National Comprehensive Cancer Network (NCCN) guidelines call for the use of radiation therapy in patients with suspected or proven T3‐T4 disease and/or regional lymph node involvement.^67^ Kunneman et al. conducted a four‐round Delphi study on the benefits/harms of preoperative radiation therapy and found poor congruence between topics important to patients and those addressed by oncologists in daily practice. Specifically, male patients believed that erectile dysfunction, ejaculatory function, and infertility should be addressed during consultation, while female patients found the topics of vaginal dryness, dyspareunia, menopause, and infertility important.^68^

In summary, radiation therapy poses a significant risk for worsened sexual function independently of surgical considerations. All patients should be counseled on these risks when considering radiotherapy in treatment plan discussions. Additionally, efforts should be made to minimize the non‐essential use of radiotherapy for rectal cancer unless clinically indicated.

## RESTORATION OF ERECTILE FUNCTION

Erectile dysfunction is defined as the inability to achieve and/or maintain an erection that is suitable for satisfactory sexual performance.^69^, ^70^ ED is diagnosed through extensive clinical questionnaires, Doppler ultrasounds, and laboratory measurements.^69^ There is debate as to how long the dysfunction needs to persist to meet criteria for ED. There are various causes of ED; however, here we will focus on therapies related to damage caused by surgery for colorectal cancer.

### Phosphodiesterase‐5 inhibitors

As erectile and ejaculatory dysfunction are potential complications associated with both (APR) and LAR, it is important to understand treatment options for attempting to restore sexual function (Table 2). Using a PDE5i increases intracellular cGMP levels and allowing for a prolonged erection.^71^ Aside from sildenafil, other FDA‐approved potent PDE5is include tadalafil, avanafil, and vardenafil, with tadalafil becoming increasingly popular.^71^, ^72^, ^73^ Compared to these other approved PDE5is, sildenafil shows increased peak systolic velocity, improving penile circulation long‐term.^74^ Compounded with libido stimulation, PDE5is may result in better erectile recovery.^75^

**TABLE 2:** Pharmaceutical agents for the treatment of erectile and ejaculatory dysfunction as a complication of abdominoperineal resection or low anterior resection.

Treatment Class	Treatment Name	Mechanism of Action	Pharmacokinetic Properties	Indications	Contraindications	Potential Side Effects
Phosphodiesterase‐5 inhibitors	Sildenafil	Inhibits PDE5, increases intracellular cGMP levels, leads to erection	Time to Peak Concentration: 60 min; Half‐life: 3–4 h	Erectile dysfunction	Concomitant use with nitrates due to exacerbation of hypotensive effects, Peyronie's disease, bleeding disorder, retinitis pigmentosa, stroke, severe heart or liver problem, multiple myeloma, sickle cell anemia, allergy	Headaches, nausea, flushing, indigestion, epistaxis, mild decrease in blood pressure, priapism
	Tadalafil		Time to Peak Concentration: 120 min; Half‐life: 18–36 h			
	Avanafil		Time to Peak Concentration: 30 min; Half‐life: 5 h			
	Vardenafil		Time to Peak Concentration: 60 min; Half‐life: 4–5 h			
Intracavernosal Injections	Alprostadil	PGE1 analog, increases intracellular cAMP, leads to erection	Time to Peak: 30 min; Half‐life: 1 h; 81% bound to albumin	Erectile dysfunction	Known hypersensitivity, sickle cell anemia, polycythemia vera, multiple myeloma, Peyronie's disease, thrombocytopenia	Cyanosis, chest pain, fever, skin redness, bradycardia, arrhythmia
	Papaverine	Inhibits PDE5, increases intracellular cGMP, non‐specific calcium channel blocker, promotes penile vasodilation, leads to erection	Time to Peak Concentration: 30 min, Half‐life: 2–4 h	Erectile dysfunction	Glaucoma, heart block, sinus tachycardia, hepatic or renal impairment, allergy	Flushing, headache, dizziness, skin rash, diarrhea, stomach pain
	Phentolamine	Penile smooth muscle alpha‐1 blocker, decreases smooth muscle tone, increased vasodilation, increases NO release from nerve terminals, leads to erection	Time to Peak Concentration: 5–10 min, Half‐life: 0.5–2 h	Erectile dysfunction	Hypersensitivity to drug, concomitant use with other alpha‐adrenergic blockers, myocardial infarction, angina, coronary artery disease	Tachycardia, orthostatic hypotension, weakness, hypotensive episodes, flushing, dizziness, nausea
	Vasoactive Intestinal Peptide (VIP)	Binds to VIP receptors, increases cAMP levels, increases NO release from nerve terminals, leads to erection	Time to Peak Concentration: 10–15 min, Half‐life: 0.5–2 h	Erectile dysfunction	Hypersensitivity to drug, pancreatitis	Flushing, heart palpitations, nausea, headache
	Atropine	Blocks ACh, blocks vasoconstriction, increases vasodilation, leads to erection	Time to Peak Concentration: 20 min, Half‐life: 2–4 h	Bradycardia, irritable bowel syndrome, typically not used for erectile dysfunction	Glaucoma, pyloric stenosis, fever, thrytoxicosis, obstructive uropathy	Mydriasis, vision problems, chest pain, allergic reaction, arrythmia, dry mouth
	BiMix	Uses synergistic effects of drugs combined, leads to erection	Time to Peak Concentration: 5–20 min, Half‐life: 1–3 h	Erectile dysfunction	Hypersensitivity, other contraindications based on components of drug (papaverine and phentolamine for both, prostaglandin E1 also for TriMix)	Redness, swelling, pain, bleeding, dizziness, priapism, lumps
	TriMix					
Alpha‐1 sympathomimetics	Midodrine	α1 receptor agonist, assists in transurethral bladder neck closure, induces antegrade stimulation via sympathetic nerves	Time to Peak Concentration: 30–60 min, Half‐life: 3–4 h	Orthostatic hypotension, anejaculation	Hypersensitivity, acute renal disease, pheochromocytoma, occlusive vascular disease, bradycardia, arrhythmia, thyrotoxicosis	Blurry vision, headache, dizziness, itching. rash
	Ephedrine		Time to Peak Concentration: 1–2 h, Half‐life: 3–6 h	Nasal decongestion, anejaculation	Hypersensitivity, severe hypertension, tachycardia, arrhythmia, coronary artery disease, angina, hyperthyroidism, glaucoma, diabetes, pheochromocytoma, prostatic hypertrophy	Blurry vision, headache, dizziness, tachycardia, arrhytmia itching, arrthymia, rash
	Phenylpropanolamine		Time to Peak Concentration: 2–4 h, Half‐life: 5–20 h	Nasal decongestion, anejaculation	Hypersensitivity, severe hypertension, tachycardia, arrhythmia, coronary artery disease, angina, hyperthyroidism, glaucoma, diabetes, pheochromocytoma, prostatic hypertrophy	Hypertension, headache, vomiting, diarrhea, appetite, increased thirst, seizures
	Pseudoephedrine		Time to Peak Concentration: 1–2 h, Half‐life: 5–20 h	Nasal decongestion, anejaculation	Hypersensitivity, severe hypertension, tachycardia, arrhythmia, coronary artery disease, angina, hyperthyroidism, glaucoma, diabetes, pheochromocytoma, prostatic hypertrophy	Headache, dry mouth, shortness of breath, arrhythmia
	Imipramine		Time to Peak Concentration: 2–4 h, Half‐life: 12–24 h	Depression, enuresis, anejaculation, fibromyalgia, panic disorder	Hypersensitivity, recent myocardial infarction, severe hepatic disease, glaucoma, urinary obstruction, prostatic hypertrophy, use of monoamine oxidase inhibitors in past 14 days	Vision problems, cold sweats, dry mouth, fatigue, stomach pain, urinary changes

### Intracavernosal injections

In addition to PDE5is, intracavernosal injections (ICIs) have been utilized in the treatment of (ED), the most common of which are injections of intracavernosal alprostadil. Functionally, alprostadil, a synthetic analog of prostaglandin E1 (PGE1), binds to G‐coupled PGE1 receptors on smooth muscle cell surfaces.^76^ Before this treatment option is selected, physicians should consider patients' potential fear of penile injections. Both PDE5i and ICI with alprostadil were found to be efficacious and well‐endured.^77^

### Inflatable penile prosthesis

Apart from PDE5is and ICIs, another therapeutic alternative that exists is the inflatable penile prosthesis (IPP). Over the years, several engineering improvements have been made to the device, and one recent study found that the median device survival of an IPP is about 20 years.^78^

Notably, a challenge that surgeons face with IPP surgery is implant reservoir placement. Surgeon preference dictates reservoir placement, with the most common being in the space of Retzius (SoR); the extraperitoneal space between the pubic symphysis and the urinary bladder.^79^ However, in the case of prior violation of the SoR, reservoir placement in that location increases the risk of bladder injury. Alternative reservoir placements may be considered, including high or low submuscular, with or without the use of a counter incision.^80^, ^81^, ^82^

While IPP placement is typically safe, intraoperative complications that can occur are proximal corporal perforation; distal corporal perforation; urethral injury; corporal crossover; and bowel, bladder, or vascular injury.^83^ Postoperatively, complications that can occur are reservoir herniation, reservoir erosion, auto‐inflation, implant infection, mechanical failure, hematoma, and venous compression syndrome, although the incidence of these complications is quite low.^83^, ^84^, ^85^ As IPPs are more invasive than other treatment options and associated with surgical risks, this approach should be reserved for ED patients who have not responded to other treatments, which may be due to comorbidities such as diabetes, severe penile fibrosis, or Peyronie's disease.^78^

## RESTORATION OF EJACULATORY FUNCTION

### Retrograde ejaculation

For restoration of ejaculatory function post‐APR or post‐LAR, retrograde ejaculation must first be ruled out. Treatments for retrograde ejaculation have been studied extensively. Electroejaculation for management of retrograde ejaculation has helped result in successful pregnancies, although the numbers are small.^86^ The reason electroejaculation may be unfruitful is that it is associated with poorer semen quality compared to other techniques such as penile‐vibro stimulation (PVS).^87^

Another approach for treating retrograde ejaculation involves alkalization of urinary pH to preserve sperm viability.^88^ Namely, retrograde ejaculation can be treated using sodium bicarbonate, sodium citrate, or potassium bicarbonate, as the acidity and osmolarity of urine has toxic effects on sperm.^89^ Additionally, there has been success in restoring antegrade ejaculation in patients by injecting Deflux, a gel composed of dextranomer and hyaluronic acid typically used to treat vesicoureteral reflux in pediatric patients, into the transurethral bladder neck.^90^, ^91^ Intrauterine insemination, in vitro fertilization, and intracytoplasmic sperm injection have also proven to be successful in the treatment of retrograde ejaculation.^92^ To aid in this process, urinary sperm retrieval can be done by first emptying and washing the bladder with Ringer's lactate or sperm wash medium using catheterization.^93^, ^94^ Then, Ringer's lactate or sperm wash medium is instilled into the bladder before the catheter is removed.^93^, ^94^

### Anejaculation

Anejaculation refers to the condition in which a person is unable to ejaculate any semen during sexual activity, which could be due to reasons such as nerve damage, pharmaceutical side effects, or previous surgery. For sperm retrieval in patients with anejaculation, penile vibratory simulation (PVS) or electroejaculation is utilized.^95^, ^96^ Typically, these stimulations have been used for patients with spinal cord injuries with a completely intact ejaculatory reflex arc,^97^, ^98^, ^99^ and the best outcomes have resulted for those who have complete upper motor lesions above the T10 level.^100^, ^101^ When choosing between types of vibrators, vibrator outputs with regard to frequencies and peak‐to‐peak amplitudes should be considered.^102^ With regard to post‐LAR or post‐APR patients with anejaculation problems, PVS might not be the preferred option, as this is uncomfortable in sensate patients. In such patients electroejaculation, which can be performed under general anesthesia, is likely the more appropriate option. Dopaminergic drugs such as oxytocin or clomiphene citrate may be used to improve ejaculatory nerve sensitivity or semen production.^103^, ^104^

## CONCLUSION

APR and LAR are valuable surgical treatment options for rectal cancer. However, complications from these surgeries can result in sexual dysfunction, specifically including erectile and ejaculatory dysfunction for male patients. Preventively, more studies must be conducted on pelvic autonomic nerve‐sparing approaches in APR and LAR. Additionally, it is vital for rectal cancer patients to be well informed about treatment options for surgical‐related ejaculatory and erectile dysfunction as preserving sexual function may be important to a patient's QoL.

## AUTHOR CONTRIBUTIONS

**Armin Ghomeshi:** Conceptualization; methodology; visualization; writing – original draft; writing – review and editing. **John Zizzo:** Investigation; writing – original draft; writing – review and editing. **Raghuram Reddy:** Writing – original draft; writing – review and editing. **Joshua White:** Writing – original draft; writing – review and editing. **Aden Swayze:** Writing – original draft. **Sanjaya Swain:** Methodology; writing – review and editing. **Ranjith Ramasamy:** Conceptualization; resources; supervision; visualization; writing – review and editing.

## CONFLICT OF INTEREST STATEMENT

None.
